# Supplementary material for: ChromaFold predicts the 3D contact map from single-cell chromatin accessibility
Source: bioRxiv. 2023 Jul 28:2023.07.27.550836. Preprint. [Version 1] doi: 10.1101/2023.07.27.550836 (PMC10402156; doi:10.1101/2023.07.27.550836)
Supplement: 2 [file NIHPP2023.07.27.550836v1-supplement-2.pdf]

bioRxiv preprint doi: https://doi.org/10.1101/2023.07.27.550836; this version posted July 28, 2023. The copyright holder for this preprint (which was not certified by peer review) is the author/funder, who has granted bioRxiv a license to display the preprint in perpetuity. It is made available under aCC-BY 4.0 International license.

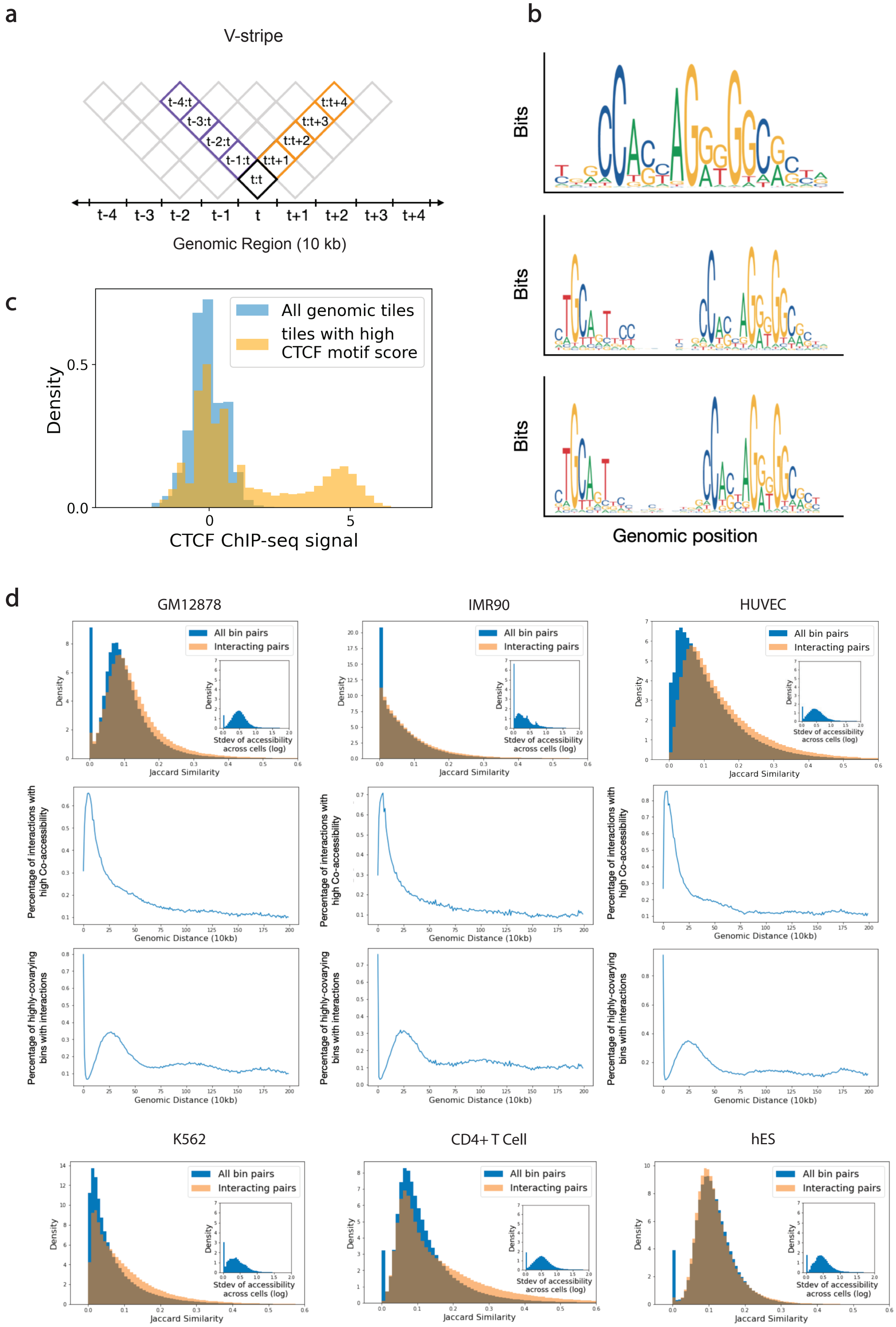

bioRxiv preprint doi: https://doi.org/10.1101/2023.07.27.550836; this version posted July 28, 2023. The copyright holder for this preprint (which was not certified by peer review) is the author/funder, who has granted bioRxiv a license to display the preprint in perpetuity. It is made available under aCC-BY 4.0 International license.

Supplementary Figure 1. ChromaFold model choices and input analysis. a. ChromaFold's prediction scheme for the chromatin interaction map. Each diamond *t_i*:*t_j* represents the interaction between genomic bins *t_i* and *t_j*. For each input centered around tile *t*, ChromaFold predicts the interaction between tile *t* and its neighboring bins within 2Mb. **b.** CTCF motif PWMs used for CTCF motif scoring. **c.** Histogram shows the distribution of CTCF ChIP-seq signal in genomic bins with top 0.1% CTCF motif score (yellow) and in all genomic bins (blue). **d.** Analysis of the extent of overlap between Hi-C interaction and Jaccard similarity in training cell types. (Top) Histogram show the distribution of Jaccard similarity between interacting bin pairs (top 10% HiC-DC+ Zscore; orange) and all tile pairs within a 2Mb distance (blue). The embedded histogram shows the variability of ATAC-seq accessibility across cells. (Middle) The line plot shows the percentage of interacting bin pairs with high Jaccard similarity (top 10%) at each genomic distance. (Bottom) The line plot shows the percentage of bins with high Jaccard similarity that are interacting at each genomic distance.

bioRxiv preprint doi: https://doi.org/10.1101/2023.07.27.550836; this version posted July 28, 2023. The copyright holder for this preprint (which was not certified by peer review) is the author/funder, who has granted bioRxiv a license to display the preprint in perpetuity. It is made available under aCC-BY 4.0 International license.

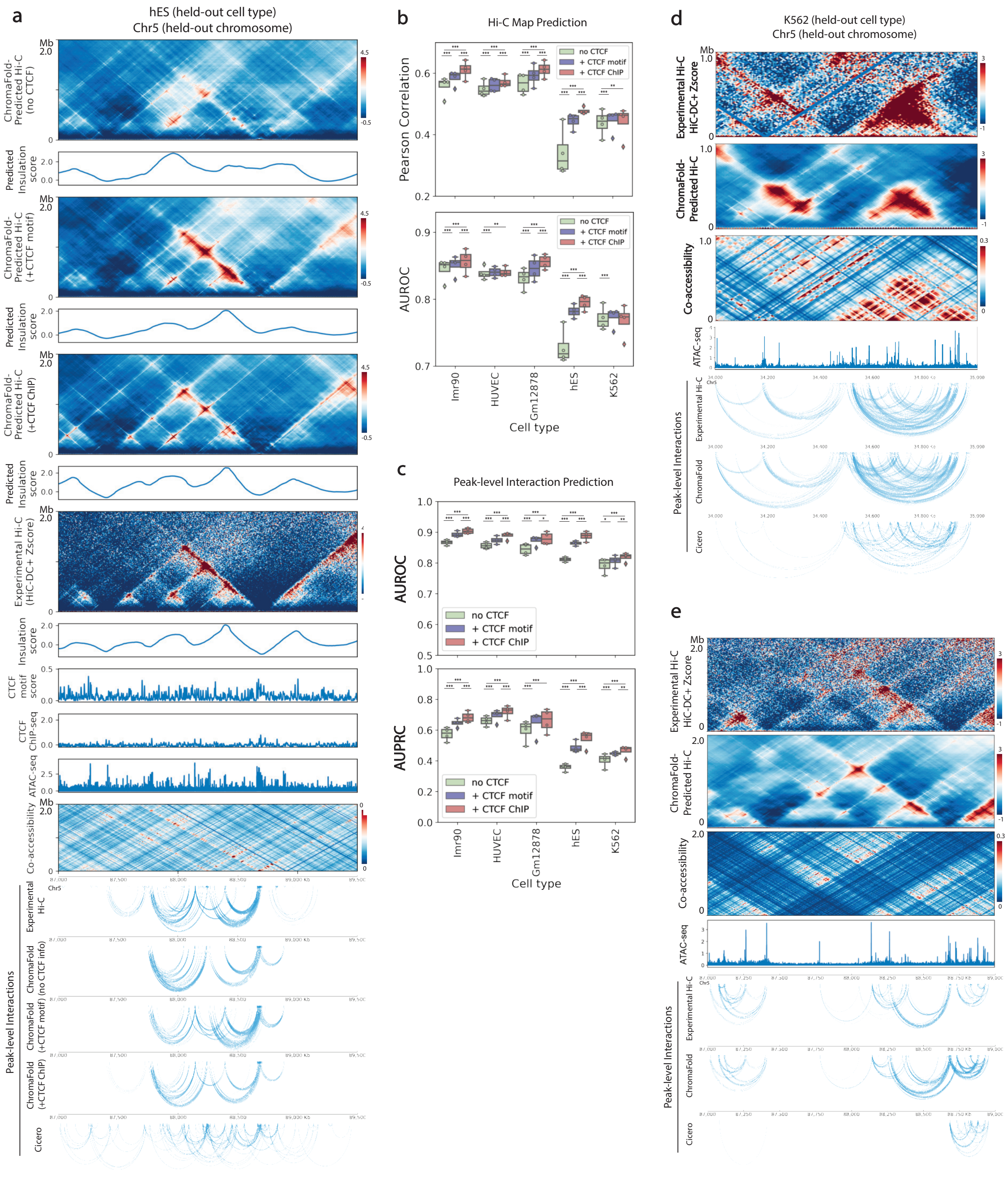

Supplementary Figure 2. CTCF information is crucial for accurate prediction of Hi-C interactions. a. Visualization of Hi-C contact maps, insulation scores and peak-level interactions predicted by ChromaFold using no CTCF information, CTCF motif score and CTCF ChIP-seq data as input in held-out cell type hESC on held-out chromosome 5. **b.** Box plots show (top) the averaged distance-stratified Pearson correlation between the experimental and predicted contact

bioRxiv preprint doi: https://doi.org/10.1101/2023.07.27.550836; this version posted July 28, 2023. The copyright holder for this preprint (which was not certified by peer review) is the author/funder, who has granted bioRxiv a license to display the preprint in perpetuity. It is made available under aCC-BY 4.0 International license.

map and (bottom) the averaged distance-stratified AUROC of significant interactions (top 10% in Z-score), per held-out chromosome. Paired t-test is performed on the distance-stratified person correlation across test chromosomes (P-value *: <0.05, **: < 0.01, ***: < 0.001). **c.** Box plots show the AUPRC (top) and AUROC (bottom) of significant peak-level interaction prediction per held-out chromosome. Statistical test is the same as above. **d, e.** Additional visualization of ChromaFold-predicted Hi-C contact map and significant peak-level interactions and Cicero-predicted peak-level interactions in held-out cell type K562 on held-out chromosomes.

bioRxiv preprint doi: https://doi.org/10.1101/2023.07.27.550836; this version posted July 28, 2023. The copyright holder for this preprint (which was not certified by peer review) is the author/funder, who has granted bioRxiv a license to display the preprint in perpetuity. It is made available under aCC-BY 4.0 International license.

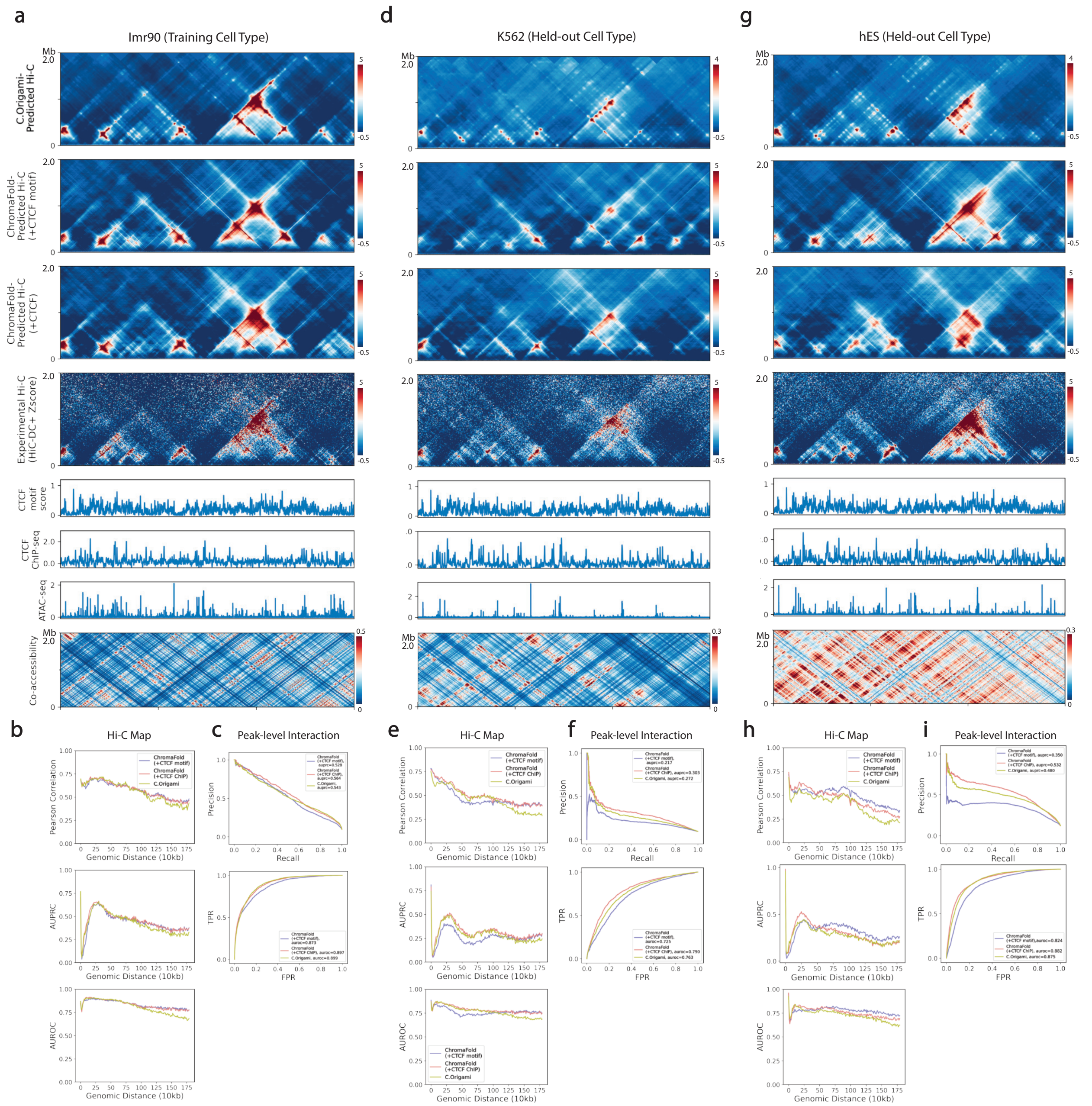

Supplementary Figure 3. ChromaFold outperforms C.Origami at prediction of HiC-DC+ normalized contact maps in held-out cell types. Visualization of C.Origami and ChromaFold predictions in training cell type IMR-90 (**a, b, c**), held-out cell type K562 (**d, e, f**) and hESC (**g, h, i**): HiC-DC+ normalized Hi-C contact maps (**a, d, g**); distance-stratified Pearson correlation for Hi-C contact map prediction, distance-stratified AUPRC and AUROC of significant interactions (top 10% in Z-score) (**b, e, h**); and PR and ROC curve for peak-level interaction prediction (**c, f, i**).

bioRxiv preprint doi: https://doi.org/10.1101/2023.07.27.550836; this version posted July 28, 2023. The copyright holder for this preprint (which was not certified by peer review) is the author/funder, who has granted bioRxiv a license to display the preprint in perpetuity. It is made available under aCC-BY 4.0 International license.

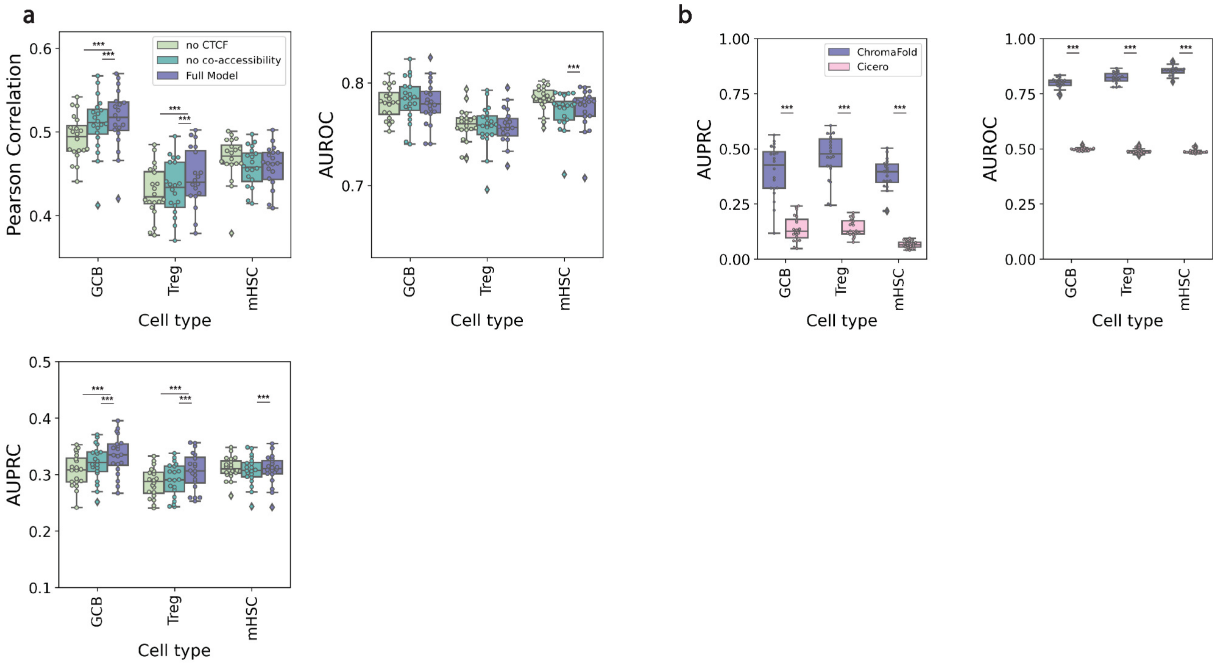

Supplementary Figure 4. Quantitative evaluation and mode comparison in mouse cell types. a. Comparison between ChromaFold model performance in the absence of CTCF motif score information and co-accessibility information. Box plots show the averaged distance-stratified Pearson correlation between the experimental and predicted contact map and the averaged distance-stratified AUPRC and AUROC of significant interactions (top 10% in Z-score), per chromosome. Paired t-test is performed on the distance-stratified person correlation across test chromosomes (P-value *: <0.05, **: < 0.01, ***: < 0.001). **b.** Comparison between ChromaFold and Cicero. Box plots show the AUPRC (top) and AUROC (bottom) of significant peak-level interaction prediction per held-out chromosome. Statistical test is the same as above.

bioRxiv preprint doi: https://doi.org/10.1101/2023.07.27.550836; this version posted July 28, 2023. The copyright holder for this preprint (which was not certified by peer review) is the author/funder, who has granted bioRxiv a license to display the preprint in perpetuity. It is made available under aCC-BY 4.0 International license.

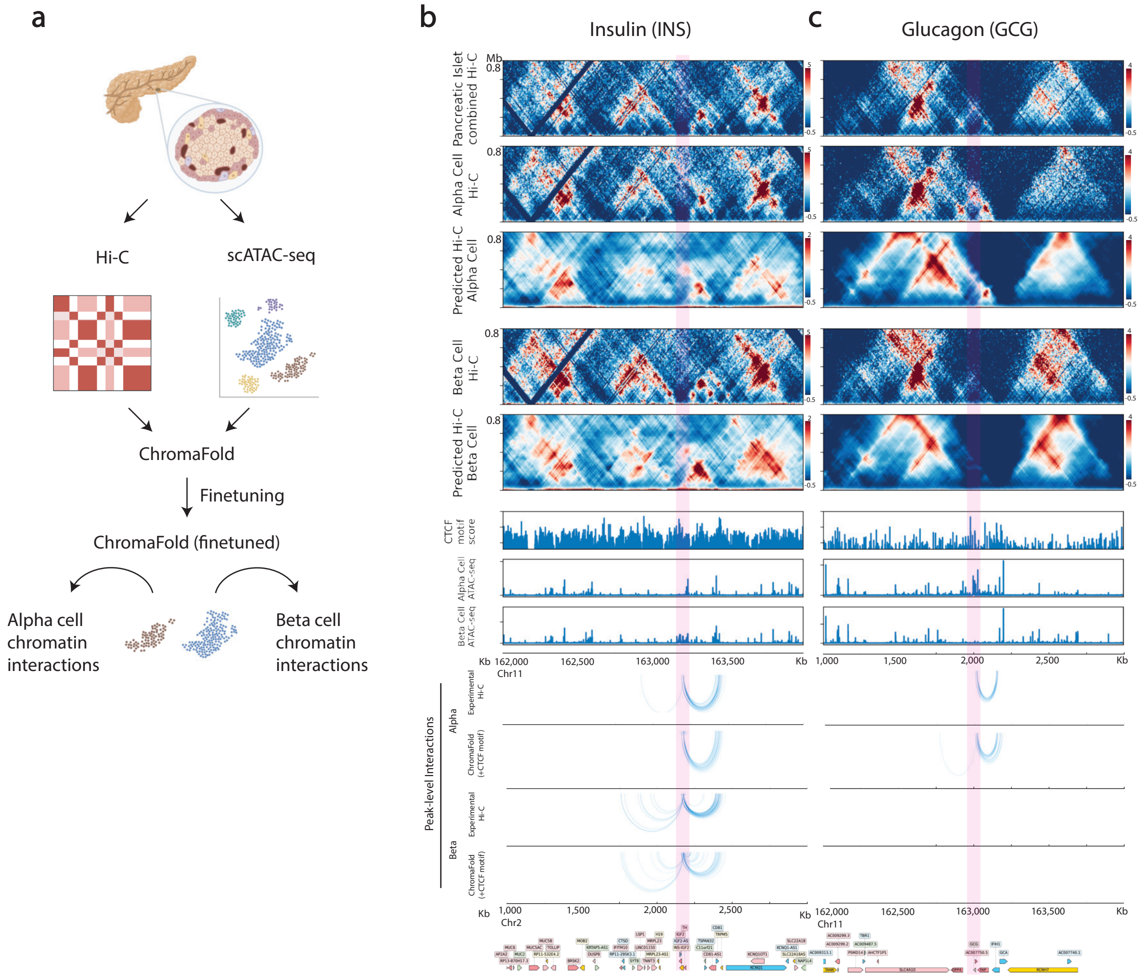

Supplementary Figure 5. Deconvolution of pancreatic islet cell contact maps at additional loci. a. Schematic of ChromaFold applied to the task of deconvoluting chromatin interactions in a complex tissue. **b, c.** Visualization of deconvolved contact maps (top) and peak-level interactions (bottom) in alpha cells and beta cells near the TSS of (**b**) glucagon (*GCG*), and (**c**) insulin (*INS*).
